# Stereotactic body radiotherapy (SBRT) re-irradiation for local failures following radical prostatectomy and post-operative radiotherapy

**DOI:** 10.1007/s00066-023-02187-2

**Published:** 2023-12-29

**Authors:** Wojciech Majewski, Marcin Miszczyk, Donata Graupner, Bartłomiej Goc, Gregor Goldner, Aleksandra Napieralska

**Affiliations:** 1 Radiotherapy Department, Maria Sklodowska-Curie National Research Institute of Oncology, Wybrzeże Armii Krajowej 15, 44-100 Gliwice, Poland; 2 III Department of Radiotherapy and Chemotherapy, Maria Sklodowska-Curie National Research Institute of Oncology, Wybrzeże Armii Krajowej 15, 44-100 Gliwice, Poland; 3 Department of Radiation Oncology, Comprehensive Cancer Center, Medical University of Vienna, Spitalgasse 23, 1090 Vienna, Austria

**Keywords:** Reirradiation, Prostate cancer, SBRT, Salvage, Postprostatectomy, Local failure

## Abstract

**Purpose:**

Local recurrences after radical prostatectomy (RP) and postoperative radiotherapy (RT) are challenging for salvage treatment. Retrospective analysis of own experiences with salvage re-irradiation was performed.

**Methods:**

The study included all consecutive patients treated with salvage stereotactic body radiotherapy (sSBRT) for prostate bed recurrence following RP and postoperative RT at a single tertiary center between 2014 and 2021. Treatment toxicity defined as the occurrence of CTCAE grade ≥ 2 genito-urinary (GU) or gastro-intestinal (GI) adverse events (AEs) was assessed. A PSA response, biochemical control (BC) and overall survival (OS) were also evaluated.

**Results:**

The study group included 32 patients with a median age of 68 years and a median follow-up of 41 months, treated with CyberKnife (53%) or Linac (47%) sSBRT. Total dose of 33.75–36.25 Gy in five fractions (72%) was applied in the majority of them. Approximately 19% patients reported grade ≥ 2 GU AEs both at baseline and at three months, and grade ≥ 2 GI toxicity increased from 0% at baseline to 6% at three months after sSBRT. There was some clinically relevant increase in late toxicity with 31% patients reporting late ≥ 2 GU, and 12.5% late ≥ 2 GI AEs. Two grade 3 AEs were recorded: recto-urinary fistulas. The majority of patients showed a PSA response (91% at one year post-sSBRT). The 3‑year BC was 40% and 3‑year OS was 87%.

**Conclusions:**

Manageable toxicity profile and satisfactory biochemical response suggest that SBRT in patients with local recurrence following RP and postoperative RT might be a salvage option for selected patients.

**Supplementary Information:**

The online version of this article (10.1007/s00066-023-02187-2) contains supplementary material, which is available to authorized users.

## Introduction

Clinical failures following radical prostatectomy (RP) remain a significant clinical problem. Postoperative radiotherapy (RT) is often administered as adjuvant RT in cases of high-risk characteristics found in a post-prostatectomy specimen or, more commonly, as salvage RT in cases of a biochemical failure. Following postoperative RT, it is still expected that 30–40% of patients will develop a biochemical failure, with local failures in up to 10% of the cases [1–4]. Considering the large number of patients treated for prostate cancer worldwide, local failures after post-prostatectomy radiotherapy are important problem. Contrary to local failures following definitive RT, there is no sufficient level of evidence for local salvage RT following postoperative RT, and the available data is limited to small and heterogeneous study groups with relatively short follow-up.

Due to the potential sequelae of two major previous interventions inside the prostate bed, any salvage treatment presents a major clinical challenge. The selection criteria for optimal candidates for repeated local salvage treatment are not well established. Among the available modalities, stereotactic body radiotherapy (SBRT) could be an option, similar to local salvage following definitive RT, for which either high-dose-rate brachytherapy or SBRT provided a comparable outcome [5]. However, the data on post-prostatectomy re-irradiation is scarce, and the largest pooled analysis of SBRT was based on only a little more than 100 patients [6]. Also, the recently published multicenter retrospective analysis reported the outcome of only 117 patients [7]. The unanswered questions regarding the required diagnostic imaging, histopathologic confirmation of the recurrence, optimum dose, fractionation, and tolerance doses for organ-at-risk or irradiated volume were raised by the editors after that publication [8].

In our institution we have used SBRT in cases of locally relapsing prostate cancer following RP and post-operative RT, and the aim of the study was to summarize our current experience with that treatment method.

## Material and methods

### Material

The retrospective study included all consecutive patients treated with radical salvage stereotactic body radiotherapy (sSBRT) for prostate bed recurrence following RP and postoperative RT for prostate cancer (PCa). Patients received sSBRT at a single tertiary center between 2014 and 2021. After primary treatment, patients were usually followed by a urologist and radiation oncologist. In cases of local failure, they were referred to salvage SBRT (salvage surgery was not the usual policy). If the patient was suitable for local salvage therapy, a decision on reirradiation (sSBRT) was made.

The diagnosis of oligometastases was not considered an exclusion criterion in patients who received previously or simultaneously metastases-directed therapy (MDT). Concurrent androgen-deprivation therapy (ADT) was allowed. The decision regarding ADT was left to the attending physician; however, patients were often given hormonal therapy before referral to salvage re-irradiation.

Histopathological confirmation of prostate bed recurrence was not mandatory in patients with rising PSA concentration and unequivocal lesions on medical imaging (MRI and/or PSMA-PET). Two patients lost to follow-up shortly after the completion of sSBRT without any data on treatment toxicity or outcome were excluded from the analysis.

### Methods

The primary endpoint was treatment toxicity, defined as the occurrence of grade 2 or higher genitourinary (GU) and gastrointestinal (GI) adverse events (AEs) according to the CTCAE v5.0 scale. The events were categorized as baseline if reported before sSBRT initiation, early if occurring up to three months after sSBRT, or late thereafter. The maximum toxicity was scored irrespective of whether the symptoms resolved or persisted. All AEs were reported. However, symptoms were considered attributable to sSBRT only when they occurred or worsened after sSBRT as compared to those already present before sSBRT.

The secondary endpoints included efficacy defined as biochemical response (BR), biochemical control (BC) and overall survival (OS).

The BR was defined as any PSA decrease after sSBRT or, in the case of an undetectable PSA level, its maintenance, assessed at various time points during the follow-up. The BC was calculated from the last day of sSBRT to the event of PSA exceeding an absolute value of 0.2 ng/ml, or censored at the last known date of PSA testing. The cut-off point of 0.2 ng/ml is the same as the AUA definition of biochemical failure after prostatectomy. We have chosen that because we believe that in a post-prostatectomy setting, PSA should drop to an undetectable level or at least 0.2 ng/ml if a durable cure is expected. Finally, OS was calculated from the last day of sSBRT until death from any cause, or censored at the last known time point at which the patient was still alive.

### Statistical analysis

Comparisons between groups for the binary variable were performed using Fisher’s exact test. Continuous variables were compared using the Mann-Whitney test, assuming a non-normal distribution for all variables. The time-to-event curves (BC and OS) were estimated using the Kaplan-Meier method, and groups were compared with log-rank testing. The *p*-values of 0.05 or lower were considered statistically significant.

## Results

This retrospective study included 32 consecutive patients treated with sSBRT for local PCa recurrence inside the prostate bed. The median age was 68 years (IQR: 65.5–74), and the median follow-up was 41 months. All patients had previously undergone RP and subsequent adjuvant (22%) or salvage (78%) RT. Post-operative radiotherapy included elective pelvic lymph node irradiation in 9 cases (28%), and the majority received at least 66 Gy to the prostate bed (81%). The detailed information on primary and sSBRT fractionation schedules is presented in a Supplementary File. The median time between RP or postoperative RT and a diagnosis of local recurrence was 98 months (IQR: 66–155) and 68 months (IQR: 44–100), respectively. The diagnosis of local recurrence was based on MRI in 10 patients (31%) and/or PET-CT in 28 patients (87.5%). A biopsy of a recurrent tumor was performed in 13 cases (41%); in one case, it was negative and in the remaining cases, recurrent cancer was pathologically confirmed. In each case, the diagnosis of local recurrence was preceded by a biochemical recurrence (PSA > 0.2 ng/ml). The median pre-SBRT PSA concentration was 1.33 ng/ml (IQR: 0.49–3.28, range: 0.01–39). Nine patients (28%) had earlier or synchronous oligometastatic disease treated with Metastases-Directed Therapy (MDT). Half of the patients in the whole group received ADT concurrently with sSBRT. The detailed study group characteristics are presented in Table 1.

**Table 1:** Clinical characteristics of the patients treated with salvage re-irradiation for local prostate bed recurrence following radical prostatectomy and postoperative radiotherapy

Clinical factor	Number (%)
**Age-**Mean 69 years (SD ± 6.7)	
*Primary ISUP Grade*	
G1	11 (34%)
G2	7 (22%)
G3	0
G4	6 (19%)
G5	6 (19%)
Unknown	2 (6%)
*Failure type*	
Isolated local failure	23 (72%)
Local failure + oligometastases	9 (28%)
*Location of local failure*	
Retrovesical	12 (38%)
Periurethral	18 (56%)
Both	2 (6%)
*Pre-SBRT PSA concentration*	
< 0.2 ng/ml	3 (9%)
0.2–0.5 ng/ml	5 (16%)
0.5–2.0 ng/ml	13 (41%)
2.0–5.0 ng/dl	4 (12%)
> 5.0 ng/dl	7 (22%)
*Neoadjuvant/concurrent ADT*	
Yes	16 (50%)
No	16 (50%)
*Pre-SBRT hormonal status*	
HSPC	28 (88%)
HSPC progressive on antiandrogen alone	2 (6%)
CRPC	2 (6%)

*ADT* Androgen Deprivation Therapy, *HSPC* Hormone Sensitive Prostate Cancer, *CRPC* Castration Resistant Prostate Cancer

### Treatment characteristics

To ensure treatment position reproducibility, patients were immobilized in individual vacuum mattresses or in thermoplastic casts. In each patient planning, CT was performed with a slice thickness of ≤ 2 mm. A total of 25 patients (78%) had 1.5 T MRI for treatment planning. A PET-CT for treatment planning or a diagnostic one with a fusion with planning CT was performed in 28 patients (87%). In 14 patients, it was choline PET; in 15 patients, it was PSMA PET (one patient had both of them).

No special conditions for bladder filling were mandatory; however in three cases (9%), a catheter was used to control bladder filling and improve treatment reproducibility during imaging and sSBRT. Patients were recommended to empty the rectum before sSBRT. In 23 patients (72%), a fiducial marker was placed into the prostate bed, which was mandatory for CyberKnife (CK)-based sSBRT and optional for Linac-based sSBRT. As a rule, only one fiducial was placed into the prostate bed under the guidance of transrectal ultrasound. Fiducial tracking was performed in all 17 patients from the CK group and in 3 patients treated with Linac sSBRT. The remaining 3 patients from the Linac group had fiducial used only for initial positioning. In all patients’ treatment was delivered with image guidance, either KV positioning to the fiducial or CBCT.

The gross tumor volume (GTV) was defined as the recurrent lesion visible on MRI and/or PET-CT, whichever was available, fused with the treatment-planning CT. The GTV was mainly defined by DCE and DWI sequences on MRI. PSMA-PET was mainly fused to confirm the location of recurrence, and it was then intended to be enclosed within the CTV. The GTV was delineated by a radiation oncologist in cooperation with a radiologist experienced in prostate cancer imaging.

Depending on the treating physician’s discretion, no or a small (≤ 3 mm) margin was added to the GTV to form the clinical target volume (CTV). The planning target volume (PTV) was created by adding a uniform 3 mm margin around the CTV. Organs-at-risk (OARs) included the rectum, bladder, bowels, and penile bulb. Due to a lack of better literature data for re-irradiation after RP and RT, dose constraints were adapted from the Stanford protocol [9], along with the ALARA rule for all applicable OARs (As Low as Reasonably Achievable). Afterward, some dose constraints for re-irradiation after definitive RT were proposed in our center, and the same were adopted in a post-prostatectomy setting (maximum point dose in rectum and bladder ≤ 100% and ≤ 110%, D30Gy- 10% and 15%).

The fractionation schemes differed throughout the study period, reflecting the learning curve in sSBRT. Finally, it was settled that the dose would be prescribed similarly to the primary ultra-hypofractionated SBRT (5 × 7.25 Gy), then it was slightly de-escalated to 5 × 6.75 Gy, which is the currently prescribed dose at our institution. Those latter fractionation schedules were used in 72% of patients. The details are presented in a Supplementary File. Median total dose in the whole group was 33.75 Gy, median D_98_ was 34.1 Gy and median D_2_ was 37.2 Gy. SBRT was performed every other day.

The dose was planned for the PTV. The dose was specified at a specific isodose for a CyberKnife treatment (usually 85–90%) or at a 100% isodose for Linac SBRT. Seventeen patients (53%) were treated with CyberKnife and 15 patients (47%) with Linacs. Both methods could be used alternatively, but in general we started our experience with re-irradiation with CyberKnife, and Linac-based re-irradiation became more common in the later years of the study.

**Fig. 1:**
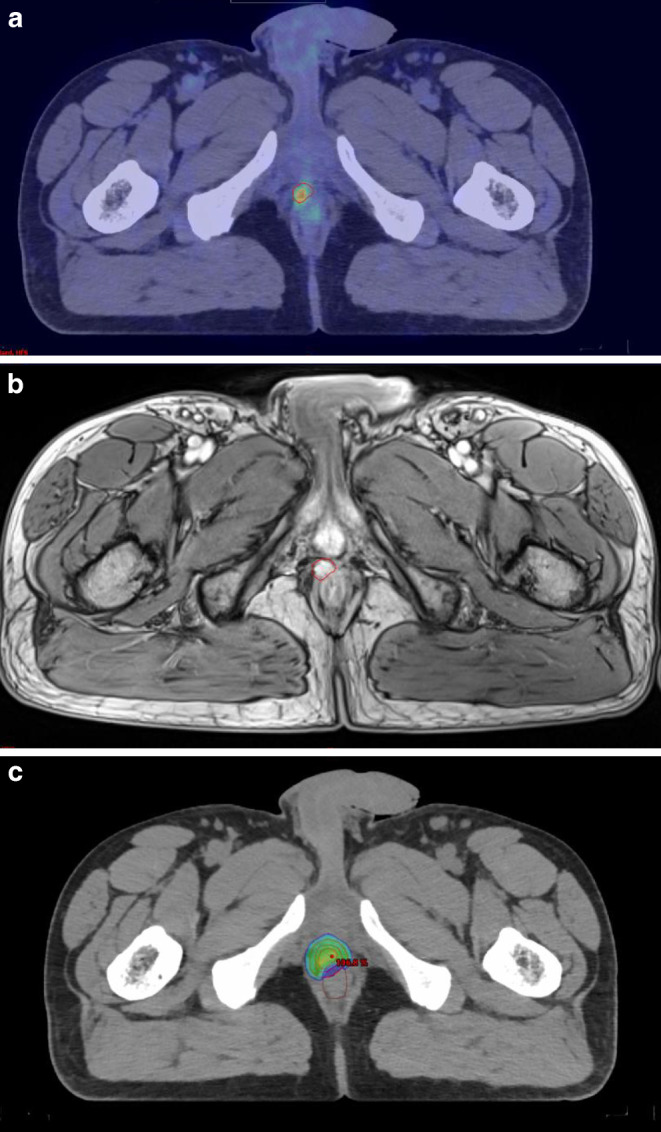
Imaging and dose distribution in an exemplary patient with local recurrence. **a** PSMA-PET: *red line* GTV, **b** MRI: *red line* GTV, **c** Dose distribution: *inner red* GTV, *middle red* CTV, *outer red* PTV, *brown* rectum

An exemplary treatment planning image and dose distribution are presented in Fig. 1a–c.

The median biologically-effective dose (BED) was 186 Gy (IQR: 186–211) assuming an α/β of 1.5 Gy for PCa. The median GTV was 2.95 (IQR: 1.65–7.25) cc, and the median PTV was 14.5 (IQR: 6.85–19.30) cc.

### Toxicity

**Fig. 2:**
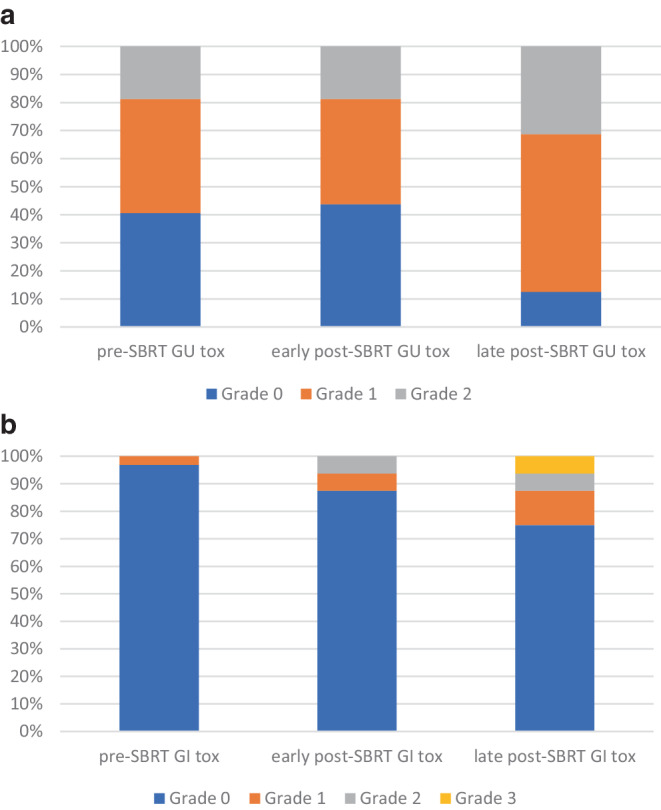
**a** Early and late GU toxicity as compared to the baseline (pre-sSBRT). **b** Early and late GI toxicity as compared to the baseline (pre-sSBRT)

Early toxicity did not significantly increase after sSBRT compared to baseline. Grade ≥ 2 GU symptoms were reported by 19% of patients, both at baseline and at in early post-SBRT period. Grade ≥ 2 GI symptoms were observed in 6% of patients (2 cases) at an early post-SBRT period, as compared to none at baseline. The differences were statistically insignificant in both aspects (GU *p* = 0.5 and GI *p* = 0.08). On the other hand, there was some increase in the occurrence of late toxicity. Late grade ≥ 2 GU AEs were reported by 31% of patients (12% increase compared to the baseline; *p* = 0.12), and grade ≥ 2 GI AEs were found in 12.5% of patients (12.5% increase compared to the baseline; *p* = 0.02). The detailed results are presented in Fig. 2a,b. There were two cases (6%) of late grade 3 AEs; two patients developed recto-urinary fistulas, which were attributable to reirradiation with SBRT. The detailed information on these patients is presented in Table 2. There was no statistically significant association between the volume of GTV or PTV and the incidence of early or late Grade ≥ 2 GU or GI toxicity.

**Table 2:** A characteristics of patients with G3 toxicity

Patient	Age	Primary RT dose	Primary RT to SBRT time	GTV PTV	Type of sSBRT	Location of the recurrence	Biopsy/ Fiducial	sSBRT Dose	Rectal DVH parameters
1	67	Salvage 66 Gy	48 months	5.5 cc 11 cc	CyberKnife	Periurethral	Yes Yes	36.25/7.25 Gy	D30%—11.2 Gy D60%—2.6 Gy Dmax—41.3 Gy
2	74	Salvage 70 Gy	100 months	9.2 cc 29.5 cc	C‑arm linac	Periurethral	No Yes	33.75/6.75 Gy	D30%—20.5 Gy D60%—6.2 Gy Dmax—33.7 Gy

### PSA response

**Fig. 3:**
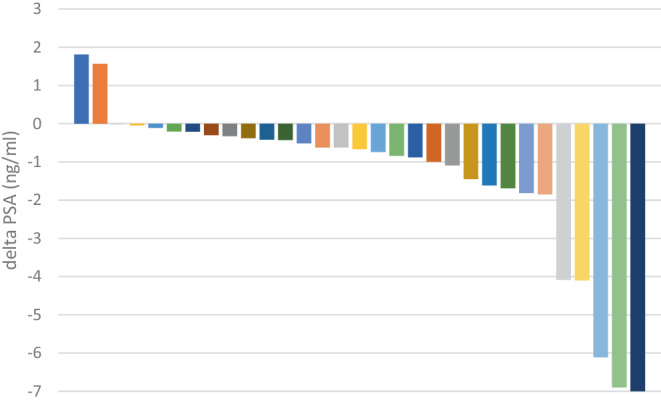
Waterfall plot of the decline of PSA (ng/ml) as compared to the pre-SBRT level at 1‑year follow-up*. *—one patient no sufficient measurement—excluded, one patient shorter than one-year—extrapolated

In the majority of cases early PSA response was observed. At one-year BR was observed in 29 patients (91% of the whole group and 94% of evaluable pts). The BR rate declined to 56% at 2‑years and 31% at 3‑years of the whole group. However, data was available only for less than two-thirds of the study group at three years. The corresponding rates in a subgroup of patients with no ADT were: 87.5% at 1‑year, 44% at 2‑years, and 19% at 3‑years. The plot of individual decline of PSA value, i.e., the absolute difference (ng/ml) as compared to the pre-sSBRT level at 1‑year, in a whole group is presented in Fig. 3.

### Long-term treatment outcome

**Fig. 4:**
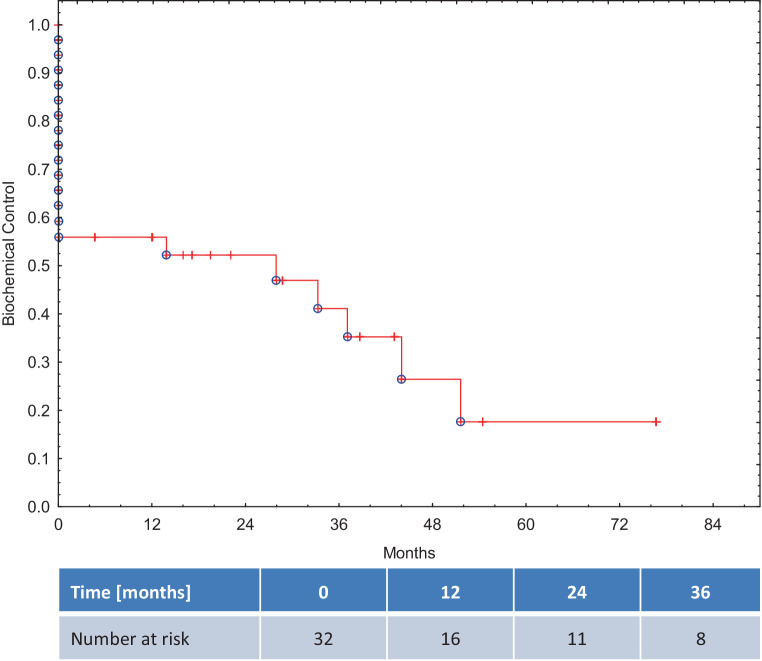
Biochemical Control in a whole study group

More than 40% of patients did not achieve a PSA decline below 0.2 ng/ml during the follow-up and were reported as having a biochemical failure from the start. Biochemical Control at 1‑, 2‑, and 3‑years was 56%, 52% and 41%, respectively. (Figure 4). In a subgroup of patients with no concomitant ADT (*n* = 16), the 1‑, 2‑ and 3‑year BC rates were 42%.

**Fig. 5:**
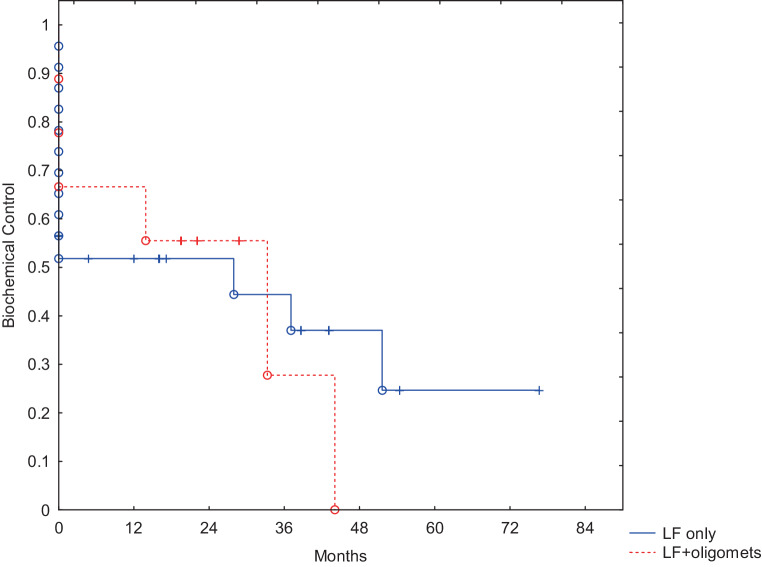
Biochemical Control in subgroups of patients with local failure alone or local failure and oligometastatic disease

We compared BC in patients with local failure only (LF) and those with oligometastatic disease (Fig. 5). Although the difference was not statistically significant, eventually no patient with concomitant oligometastatic disease remained free of biochemical recurrence.

The overall survival in a whole group at 1‑, 2‑, and 3‑years was 93%, 87%, and 87%, respectively.

## Discussion

There is no optimal treatment method for patients who have local failure following radical prostatectomy and adjuvant or salvage radiotherapy. Observation or ADT are not curative options, and local salvage therapies are underused. However, their benefits are still unclear [8]. Salvage surgery in a post-operative setting is usually not performed. Both brachytherapy and SBRT may be of interest, but the data on them is scarce in that setting. It is hypothesized that early intervention could defer the start of ADT, result in subsequent castrate resistance, or even lower the rate of metastatic progression [8]. So far, two modalities, high-dose-rate brachytherapy and SBRT, have provided similar outcomes in terms of biochemical control and late genitourinary and gastrointestinal toxicity in patients after definitive radiotherapy [5]. Salvage sSBRT in patients with local failures after RP and post-operative RT is challenging, and special attention should be given to the treatment safety and tolerance. From a theoretical point of view, a salvage treatment given after two previous major treatment modalities should be more hazardous than re-irradiation after definitive RT alone. However, the rate of severe toxicity in this study compares favorably to our experience with re-irradiation of local failures after definitive RT [10]. That favorable toxicity profile may be in part due to the lower primary radiation doses used in a post-operative setting than in a post-definitive one, possibly reducing the risk of AEs due to the lower cumulative dose. However, we think that the main reason may be attributable to the conformity of the treatment and much smaller volumes that received a high radiation dose. In the mentioned study, there was a significant difference in the toxicity between patients given focal and whole gland sSBRT (7% vs. 40% of grade ≥ 3 AEs) [10]. Whereas, in a post-prostatectomy setting, the sSBRT was always focal, directed to the macroscopic recurrence. For comparison, the median PTV in the present study was 14.5 cc, compared to 66.5 cc in sSBRT for local recurrences after definitive RT [10]. It is worth noting that focal treatment is recommended by the recently published consensus guidelines on SBRT in the post-definitive RT setting [11]. Focal re-irradiation is safer and seems quite obvious in the case of local recurrences after post-prostatectomy RT. However, Archer et al. observed that 39% of clinical relapses after re-irradiation occurred in the prostate bed outside the reirradiation volume, which raises the questions about adequate volume for re-irradiation. On the other hand, 33% in-field recurrence raises the question of possible radioresistance of the recurrence and maybe the role of image-guidance [7].

We believe that the toxicity profile in our group was acceptable, with around 12% of the patients experiencing an increase in the rate of G ≥ 2 AEs above the baseline for both GI and GU domains. The results are comparable to other studies describing re-irradiation in post-operative patients, though some of them reported even lower rates [7, 12–19]. Although we began our experience with reirradiation using lower doses (5 × 5.5 Gy, 6 × 5 Gy, etc.), we were increasing the dose to achieve a higher probability of cure. So, we used rather high doses of re-irradiation (median 5 × 6.75 Gy), compared to commonly reported 5–6 fractions of 5–6 Gy or even 18 Gy in three fractions in one of the largest series on post-prostatectomy re-irradiation [12]. De-escalation from 5 × 7.25 Gy to the dose of 5 × 6.75 Gy was recommended later, with longer follow-up of our results on sSBRT for post-definitive RT recurrences as a preventive step to avoid possible toxicity [20]. However, due to the quality of the available data, it is difficult to recommend a specific dose schedule in a post-prostatectomy scenario.

There were two concerning incidences of permanent G3 toxicity, as our study is one of the very few reporting G3 or higher AEs. However, we believe that many of the studies might be underreporting severe toxicity due to limited patients’ number and follow-up and only clinician-reported toxicity measures, and a certain rate of severe AEs in the re-irradiation setting is to be expected. For instance, one study reported a 2% incidence of G3 AEs, but did not distinguish whether they occurred in a subgroup of post-prostatectomy or post-definitive RT patients [21]. In one of the largest studies on post-prostatectomy SBRT re-irradiation, late G3 GU AEs were observed in 15% of patients, with 10% attributable to salvage SBRT [17]. In the Archer et al. study, late G3 toxicity was observed in 7 out of 117 cases, but more than 25% of patients had follow-up below 1 year [7]. One out of three patients had grade ≥ G3 GU toxicity in another study that used a similar total dose of 35 Gy in five fractions,. Importantly, it was a primary SBRT of a prostate bed without any previous RT [22].

Due to the small number of events and limited study group, we decided to omit a formal statistical analysis of prognostic factors for severe toxicity. However, some observations could be made. Patients experiencing AEs had lesions larger than the median GTV volume (2.95 cc), suggesting that the dose-volume effect may still be present even with focal treatment. It is in accordance with the data of Archer et al., who found that one of the dose-volume parameters of the bladder (D2%) and the location of a recurrence (in proximity of the urethrovesical anastomosis) were significantly associated with toxicity [7]. Hence, in patients with large tumors, a decision of salvage SBRT should be made with caution, especially if the recurrent tumor is in close proximity to the rectum or urethrovesical anastomosis, and probably it should be avoided in those with direct infiltration of the rectum or anal canal. In questionable cases, radiation schedules with a safer toxicity profile could be considered. Because of the radiobiology of prostate cancer, we would opt for SBRT in such cases, but with a reduced dose and/or more protracted fractionation. It is likely that more focus should be put on OAR dose-volume parameters, but no high-quality data on optimal dose constraints in the setting of re-irradiation is currently available [8].

A thoughtful qualification, including evaluation of previous treatment toxicity, co-morbidities, existing symptoms, and urological interventions, will probably also be essential. Treatment planning and delivery may be of importance, too. The fusion with previous treatment plans and the avoidance of possible hotspots could lower the risk of overdosage of nearby organs at risk. We believe that image guidance with cone-beam CT performed before every fraction with the evaluation of the position of the recurrence and the volume of the rectum and bladder is essential to providing a satisfactory outcome.

A one-year PSA response was observed in the majority of patients (91%). It seems comparable to the responses reported in other studies (83–90%). But, it should be stressed that in those studies, PSA response was usually reported earlier (3–6 months after sSBRT) [7, 13, 14, 16, 17]. For BC, it should be noted that there were important differences in endpoint definitions between studies. The definition used in our study (AUA definition of a biochemical failure after prostatectomy of > 0.2 ng/ml) is the most restrictive one. We have chosen that because we believe that PSA should decline to a substantially low level if a durable cure of the disease is to be expected. Nevertheless, our results compare well with most of the other literature reports presenting 2‑year BC of 30–50% [12, 15–17]. Francolini et al. reported higher rates of 2‑ and 3‑year BC, around 60%, but including both post-RP and post-RT patients and with a more liberal definition of a BC [21]. The authors observed worse BC in post-RP patients, which might reflect more aggressive histopathological features of local recurrences following RP and subsequent RT. Hence, we believe that those patient groups should be analyzed separately.

There is significant heterogeneity among available studies, and the majority of the series are small with short follow-up. An example would be the volume of irradiation; the median PTV in our group was larger than the upper limit in one of the other studies [16]. Although the pre-sSBRT PSA levels are usually comparable [7, 13, 15, 16, 19], some studies reported a significantly smaller range of PSA levels [12, 18]. Concerning treatment-related factors, Jereczek Fossa et al. have shown that BED ≥ 130 Gy is associated with better outcomes [15]. While literature data often included patients irradiated with lower BED, all except one patient in our study group were given ≥ 130 Gy BED. Finally, half of the patients in our study were receiving ADT, which is a confounding factor for BC. However, even after excluding those cases, the 3‑year BC remained above 40%, which seems quite satisfactory considering the definition of biochemical failure in the present study. The role of ADT in the sSBRT setting remains unclear; however, combining ADT with salvage postprostatectomy radiotherapy suggests some benefit from that combination [8].

Local failure is rarely the only site of the disease’s progression. We did not exclude patients with oligometastatic disease, but they all eventually experienced biochemical relapse. While some authors refer to MDT as a definitive treatment for oligometastatic disease, it is a matter of discussion if the potential toxicity of re-irradiation of non-isolated local failure is justified in such a situation.

There are still some important issues regarding optimal candidates for salvage SBRT that have not been resolved. For instance, is sSBRT suitable for patients with more aggressive disease at risk of distant metastases, like high-ISUP grade or CRPC patients?

There are several limitations to our study. Despite being one of the largest available series, the study group is small, heterogeneous, and has a relatively short follow-up as for PCa. Only in 41% of local recurrences a biopsy was performed. On the other hand, there is a huge discrepancy in the literature regarding a biopsy. In the largest multicenter analysis by Archer et al., it was even less than in our study (20%) [7]. The ESTRO-ACROP consensus did not reach agreement on the omission of a confirmatory biopsy; however, only 22% of responders considered a biopsy always necessary [11]. The salvage brachytherapy consensus endorses pre-salvage histopathological confirmation [23]. We presently accept the omission of a biopsy, but we advocate performing both MRI and PSMA-PET before the decision on reirradiation to verify the location of a recurrence with both modalities and to assess accurately for any metastatic disease.

The heterogeneity in the sSBRT regiments could also be regarded as one of the study’s limitations, but over 70% of patients received a total dose of 33.75–36.25 Gy in five fractions. Another major drawback of our study is the lack of a reliable tool to assess the local response to sSBRT. Because routine check-up visits were based on PSA measurements, MRI was not performed in the majority of patients as it was not a standard evaluation. The data were collected retrospectively, and due a to lack of a pre-defined follow-up medical imaging schedule, only BR, BC, and OS could be reliably assessed.

Finally, the treatment toxicity could be underestimated due to retrospective data collection, especially in terms of lower grade AEs. Despite that, our group was homogenous in terms of irradiated volume (only lesion-visible), and over 85% had PET-CT as part of diagnostic imaging.

To sum up, we believe that the presented results are satisfactory. The toxicity seems to be manageable, and often it is possible to achieve biochemical control. The treatment could be beneficial in well-selected patients; however, due to limited data, we were unable to define predictive factors. We think that the study adds to the experience on that challenging topic, but prospective trials or more numbered pooled analyses are necessary to verify treatment safety and efficacy and indicate optimal candidates for sSBRT.

### Supplementary Information

Supplementary File 1. Radiotherapy fractionation schedules used in the primary treatment and re-irradiation of locally recurrent prostate cancer.
