# Supplementary material for: Stereotactic body radiotherapy (SBRT) re-irradiation for local failures following radical prostatectomy and post-operative radiotherapy
Source: Strahlenther Onkol. 2023 Dec 29;200(3):230–8. doi: 10.1007/s00066-023-02187-2 (PMC10876733; doi:10.1007/s00066-023-02187-2)
Supplement: Supplementary file 1 — Radiotherapy fractionation schedules used in the primary treatment and re-irradiation of locally recurrent prostate cancer. [file 66_2023_2187_MOESM1_ESM.docx]

Supplementary File 1. Radiotherapy fractionation schedules used in the primary treatment and re-irradiation of locally recurrent prostate cancer.

| Number of patients | 1^st^ RT type | 1^st^ TD [Gy] | 1^st^ Dfx [Gy] | 2^nd^ RT type | 2^nd^ TD [Gy] | 2^nd^ Dfx [Gy] |
| --- | --- | --- | --- | --- | --- | --- |
| 2 | Salvage | 76 | 2 | SBRT | 36 | 6 |
| 1 | Salvage | 76 | 2 | SBRT | 33.75 | 6.75 |
| 1 | Salvage | 76 | 2 | SBRT | 35 | 5 |
| 1 | Salvage | 76 | 2 | SBRT | 30 | 10 |
| 1 | Salvage | 76 | 2 | SBRT | 27.5 | 5.5 |
| 1 | Salvage | 74 | 2 | SBRT | 36.25 | 7.25 |
| 2 | Salvage | 72 | 2 | SBRT | 36.25 | 7.25 |
| 1 | Salvage | 72 | 2 | SBRT | 33.75 | 6.75 |
| 1 | Salvage | 70.2 | 1.8 | SBRT | 36.25 | 7.25 |
| 1 | Salvage | 70 | 2 | SBRT | 36.25 | 7.25 |
| 4 | Salvage | 70 | 2 | SBRT | 33.75 | 6.75 |
| 1 | Salvage | 68 | 2 | SBRT | 36.25 | 7.25 |
| 1 | Salvage | 68 | 2 | SBRT | 24 | 8 |
| 1 | Adjuvant | 66.6 | 1.8 | SBRT | 36.25 | 7.25 |
| 1 | Salvage | 66 | 2 | SBRT | 36.25 | 7.25 |
| 1 | Adjuvant | 66 | 2 | SBRT | 36.25 | 7.25 |
| 1 | Salvage | 66 | 2 | SBRT | 33.75 | 6.75 |
| 2 | Adjuvant | 66 | 2 | SBRT | 33.75 | 6.25 |
| 1 | Salvage | 66 | 2 | SBRT | 30 | 6 |
| 1 | Adjuvant | 66 | 2 | SBRT | 30 | 5 |
| 1 | Salvage | 60 | 2 | SBRT | 36.25 | 7.25 |
| 1 | Adjuvant | 60 | 2 | SBRT | 36.25 | 7.25 |
| 1 | Salvage | 56 | 2 | SBRT | 33.75 | 6.75 |
| 1 | Adjuvant | 54.6 | 2.6 | SBRT | 36 | 12 |
| 1 | Salvage | 52 | 2.6 | SBRT | 33.75 | 6.75 |
| 1 | Salvage | 52 | 2 | SBRT | 33.75 | 6.75 |
| 1 | Adjuvant | 45 | 1.8 | SBRT | 36.25 | 7.25 |

RT – radiotherapy; SBRT – stereotactic body radiotherapy; TD – total dose; Dfx – fraction dose
